# *De novo* frameshift mutation in SYNGAP1 resulting in autosomal dominant mental retardation type 5 and autism spectrum disorder: a case report

**DOI:** 10.3389/fped.2025.1671464

**Published:** 2025-10-20

**Authors:** Shuangzhu Lin, Yangfan Qi, Hongyan Xie, Xiaoyu Sun, Wanqi Wang, Kai Jiang

**Affiliations:** ^1^ School of Traditional Chinese Medicine, Changchun University of Chinese Medicine, Changchun, China; ^2^ Diagnosis and Treatment Center for Children, The Affiliated Hospital of Changchun University of Chinese Medicine, Changchun, China

**Keywords:** autism spectrum disorder, global developmental delay, autosomal dominantmental retardation type 5, *SYNGAP1*, epilepsy

## Abstract

**Background:**

Autosomal Dominant Intellectual Disability Type 5 (MRD5) is caused by heterozygous mutations in the *SYNGAP1* gene. This gene, located on chromosome 6q21, encodes a synaptic Ras/Rap GTPase-activating protein that regulates Ras/Rap signaling and AMPA receptor trafficking, impacting synaptic plasticity and neuronal homeostasis. According to studies by Chen et al. and Kim et al., the *SYNGAP1* gene is localized to dendritic spines of pyramidal neurons in the rodent neocortex.

**Case summary:**

We report a 2-year-10-month-old girl presenting with global developmental delay (GDD) and autistic behaviors, characterized by unsteady gait, inability to stand on one foot, significantly impaired expressive language (maximally three-word phrases), poor response to name, reduced eye contact, and absent joint attention, despite normal hearing. Standardized assessments revealed severe impairments: Gesell Developmental Quotient (DQ) = 36, Childhood Autism Rating Scale (CARS) score = 42 (indicating severe autism), and Autism Diagnostic Observation Schedule (ADOS-2) Module 1 score = 16. Whole-exome sequencing identified a *de novo* heterozygous frameshift mutation in the *SYNGAP1* c.1230delC p. (Ser410ArgfsTer30), classified as pathogenic per ACMG guidelines. Electroencephalography (EEG) revealed no abnormalities, and brain magnetic resonance imaging (MRI) showed no structural lesions. The patient was diagnosed with MRD5 and Autism Spectrum Disorder (ASD).

**Conclusion:**

We present a case of *SYNGAP1*-related MRD5 characterized by significant global developmental delay and autism spectrum disorder, featuring a novel c.1230delC frameshift variant that has not been reported before. This discovery expands clinicians' knowledge of the mutation spectrum and phenotypic variability linked to *SYNGAP1*, enhancing understanding of genotype-phenotype relationships in *SYNGAP1*-related conditions.

## 1 Introduction

Autosomal dominant mental retardation type 5 (MRD5; OMIM #612621) is characterized by moderate to severe intellectual developmental impairment, with delayed psychomotor development manifesting in early childhood. The majority of patients experience various forms of seizures, while some exhibit autism or autism spectrum disorder, and others develop acquired microcephaly (1).

MRD5 is an uncommon neurodevelopmental disorder resulting from heterozygous mutations in the *SYNGAP1* gene (1, 2). An investigation encompassing 246 individuals diagnosed with MRD5 demonstrated that 98.7% presented with intellectual disability, 91.6% experienced epilepsy, and 57.3% exhibited symptoms consistent with Autism Spectrum Disorder (ASD) (3).

In this study, we present a case of a patient diagnosed with MRD5, characterized by significant developmental delay and autistic features, yet notably lacking seizures. Whole-exome sequencing revealed a *de novo*heterozygous frameshift mutation in the *SYNGAP1* gene, which aligns with the patient's clinical phenotype. This finding contributes to the expanding knowledge of *SYNGAP1* variants and their associated clinical presentations.

## 2 Case presentation

Chief Complaint: A 2-year-10-month-old girl was referred for evaluation of developmental delay observed over the preceding 24 months.

Two years before the evaluation, the parents observed delays in both language and motor development relative to age-matched peers; however, no systematic assessment or intervention was undertaken. The primary manifestations included an unsteady gait, inability to maintain a single-leg stance, inability to run, limited spontaneous speech, poor response to being called by name, reduced eye contact, and lack of joint attention. Expressive language was restricted to a maximum of three-word phrases. No feeding difficulties were reported, and auxological parameters, including height and weight, were within normal ranges. After admission, the patient was assessed using a standardized scale. Gesell Developmental Quotient (DQ) = 36, Childhood Autism Rating Scale (CARS) score = 42 (indicating severe autism), and Autism Diagnostic Observation Schedule (ADOS-2) Module 1 score = 16.

Physical Examination: Anthropometric assessments indicated a height of 105 cm, a weight of 14.5 kg, and a head circumference of 48 cm. Dysmorphology screening indicated the absence of dysmorphic facial features, a high-arched palate, a single transverse palmar crease (simian crease), or café-au-lait spots. Body proportions exhibited a normal upper-to-lower segment ratio. Cardiopulmonary assessment was unremarkable. Abdominal examination showed a soft, non-tender abdomen with no rebound tenderness. Neuromuscular evaluation demonstrated normal muscle strength and tone, with physiological reflexes present and no pathological reflexes observed. Spinal assessment revealed no significant scoliosis. Examination of the genitalia showed normal external genitalia without deformities.

Birth and Family History: The subject was delivered at 40 weeks of gestation through cesarean section, with a birth weight of 3.6 kg and a length of 50 cm. There were no indications of perinatal asphyxia. The maternal pregnancy history was unremarkable. There is no known family history of genetic disorders.

Routine clinical examinations: A comprehensive evaluation, including complete blood count, urinalysis, stool analysis, assessments of liver and kidney function, cardiac enzyme levels, electrolyte balance, thyroid function, and both blood and urine genetic metabolic screenings, indicated no abnormalities. Electroencephalography (EEG) revealed no abnormalities, and brain magnetic resonance imaging (MRI) showed no structural lesions.

**Figure 1:**
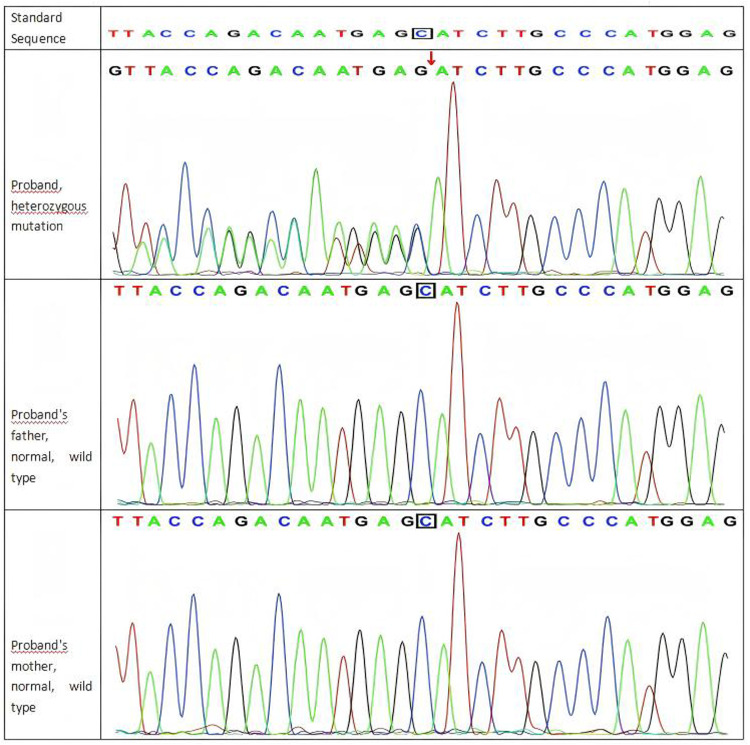
Sanger sequencing results of the *SYNGAP1* c.1230delC variant in the proband and her parents.

Genetic analysis: Whole-exome sequencing (WES) identified a *de novo*heterozygous frameshift mutation in the *SYNGAP1* c.1230delC p. (Ser410ArgfsTer30). The parents of the patient did not have this variant in the *SYNGAP1* gene. The mutation is novel (Figure 1). Its pathogenicity is confirmed per ACMG guidelines, satisfying criteria PVS1, PS2_Supporting, and PM2_Supporting.

### 2.1 Final diagnosis: MRD5, ASD

Treatment: A comprehensive rehabilitation program spanning three months was implemented for the patient.This program included not only traditional therapies (e.g., acupuncture and massage) but also weekly language training, sensory integration training, and cognitive-social skills training, which systematically improved the child's overall abilities.We also provided training for the child's parents, guiding them on family education and reinforcing therapeutic activities.

Outcome and Follow-up: The patient demonstrated significant improvements in motor function, evidenced by their ability to ambulate independently and perform bipedal hopping. In contrast, there were no observable advancements in language or social communication skills.Fortunately, the child had no seizures or any suspicious symptoms during follow-up, and no epileptiform discharges were detected by EEG.

## 3 Discussion

*SYNGAP1* is a gene that encodes the cytosolic protein SYNGAP1, an essential component of the postsynaptic density at excitatory glutamatergic neurons (4). SYNGAP1 is a negative regulator of Ras, Rap and of AMPA receptor trafficking to the postsynaptic membrane, thereby regulating not only synaptic plasticity, but also neuronal homeostasis (5).

The SYNGAP1 protein exhibits high structural and functional conservation across vertebrate species, including zebrafish and mice, and plays a critical role in early nervous system development (6, 7). This evolutionary conservation underscores not only the protein's fundamental importance in neurodevelopment but also establishes these organisms as highly relevant models for mechanistic investigation. By introducing loss-of-function mutations analogous to those found in patients, researchers can recapitulate core neurological and behavioral deficits in these models, thereby enabling direct analysis of SYNGAP1-related pathogenic mechanisms. Moreover, the experimental accessibility of these systems—such as precise genetic manipulation, real-time visualization of neural circuit dynamics, and high-throughput drug screening—provides unique opportunities to dissect underlying molecular pathways and evaluate potential therapeutic strategies that are not feasible in human subjects.

In China, cases of MRD5 attributed to mutations in the *SYNGAP1* gene have been infrequently documented. A recent investigation involving 10 children with intellectual disability and developmental delay in Shandong Province identified *de novo*mutations in the *SYNGAP1* gene. These mutations comprised five nonsense mutations, two frame-shift mutations, two splicing mutations, and one codon deletion, with five of these mutations being newly identified. These findings not only broaden the mutation spectrum of the *SYNGAP1* gene but also offer novel insights into the genotype-phenotype relationship (8).

Additionally, the study demonstrated that mutations in the *SYNGAP1* gene may result in decreased SYNGAP protein expression, a critical factor in the pathogenesis of MRD5 (8). By analyzing published *SYNGAP1* mutation cases, researchers further clarified genotype-phenotype correlations. For instance, variants in exons 1–6 may be associated with milder intellectual disability and lower autism risk, but higher likelihood of refractory epilepsy (3).

The patient described in this study initially presented with GDD and characteristics resembling Autism Spectrum Disorder. Unlike previously documented cases, this patient did not exhibit epileptic symptoms and showed normal results on brain MRI (9–15). Rehabilitation training led to a marked improvement in motor function.

Finally, we found a *de novo*heterozygous frameshift mutation (c.1230delC, p.Ser410ArgfsTer30) in the patient's *SYNGAP1* gene, which provided a clear molecular diagnostic basis for the observed neurodevelopmental phenotype and was consistent with the characteristics of autosomal dominant intellectual disability type 5 (MRD5).

The c.1230delC variant was absent in both parents, confirming its *de novo*origin. *de novo*mutations typically arise during gametogenesis or early embryogenesis, resulting in a heterozygous state in the affected individual. This mechanism fully accounts for the high heterozygosity observed at this locus. *SYNGAP1* is a known haploinsufficient gene: loss of one functional allele is sufficient to cause disease, as the remaining wild-type allele cannot compensate for the loss of function. Biallelic loss is likely embryonically lethal, which further reinforces the pathogenicity of heterozygous truncating mutations (1, 2).

The pathogenicity of this variant is robustly supported by the ACMG/AMP guidelines (16). The application of the PVS1 criterion is justified given that the frameshift mutation (c.1230delC) is highly likely to lead to nonsense-mediated mRNA decay and a complete loss of function of the SYNGAP1 protein. As *SYNGAP1* is a well-established haploinsufficiency gene, such null variants are a principal disease mechanism. This evidence is strongly corroborated by the *de novo*occurrence of the variant (PS2_Supporting) in the patient, which is a strong indicator of causality in the context of a *de novo*dominant disorder. Furthermore, the absence of the variant in public population databases (PM2_Supporting) underscores its rarity and aligns with a pathogenic role. The convergence of these evidence lines provides a high degree of confidence in the role of this variant as the primary genetic cause of the patient's condition.

It is worth noting that the child did not experience seizures, which is different from previously reported cases. We consider that this may be due to the following reasons:First, her young age raises the possibility of delayed penetrance of epilepsy, a common feature in *SYNGAP1*-related encephalopathy. Continued long-term monitoring is essential to evaluate this risk. Second, the position of the truncation may result in partial or altered functional impact compared to variants located within or C-terminal to the GAP domain. Finally, modifying genetic factors or the potential compensatory effects of early interventional therapy may also influence clinical expressivity.

Although the patient has not experienced any seizures, given the well-documented and high risk of delayed-onset epilepsy in *SYNGAP1*-related disorders, we have engaged in extensive and repeated discussions with the family. Through professional guidance, we have helped the parents recognize potential symptoms, including subtle manifestations such as staring spells, myoclonic jerks, or behavioral arrests. As of her most recent evaluation, the patient remains seizure-free. Moving forward, we have recommended EEG monitoring at six-month intervals.

We report a case of SYNGAP1-related MRD5, characterized by profound global developmental delay and Autism Spectrum Disorder, and identified by a novel c.1230delC frameshift variant not previously documented. This finding broadens the existing mutation spectrum and phenotypic variability associated with *SYNGAP1*, thereby advancing the understanding of genotype-phenotype correlations in SYNGAP1-related disorders.

## 4 Patient perspective

The patient's legal guardian provided written informed consent for the publication of this case report.

## Data Availability

The original contributions presented in the study are included in the article/Supplementary Material, further inquiries can be directed to the corresponding author.
